# Anti-cancer potency of tasquinimod is enhanced via albumin-binding facilitating increased uptake in the tumor microenvironment

**DOI:** 10.18632/oncotarget.2378

**Published:** 2014-08-21

**Authors:** John T. Isaacs, Susan L. Dalrymple, D. Marc Rosen, Hans Hammers, Anders Olsson, Tomas Leanderson

**Affiliations:** ^1^ The Sidney Kimmel Comprehensive Cancer Center at Johns Hopkins, Baltimore, MD; ^2^ The Brady Urological Institute-Department of Urology, The Johns Hopkins University School of Medicine, Baltimore, MD; ^3^ Active Biotech, AB Lund, Sweden; ^4^ Immunology group, Lund University, Sweden

**Keywords:** EPR effect, drug uptake, albumin-binding, tasquinimod

## Abstract

Tasquinimod, an orally active quinoline-3-carboxamide, binds with high affinity to HDAC4 and S100A9 in cancer and infiltrating host cells within compromised tumor microenvironment inhibiting adaptive survival pathways needed for an angiogenic response. Clinical trials document that as low as 0.5-1mg tasquinimod/day is therapeutic against castrate resistant metastatic prostate cancer. Tasquinimod is metabolized via cytochrome P4503A4, but ketoconazole at a dose which completely inhibits CYP3A metabolism does not affect tasquinimod's ability to inhibit endothelial “sprouting” *in vitro* or anti-cancer efficacy against human prostate cancer xenografts in *vivo*.

Tasquinimod's potency is facilitated by its reversible binding (K_d_ < 35 μM) to the IIA subdomain of albumin (Sudlow's site I). As blood vessels within the compromised cancer microenvironment are characterized by a higher degree of leakiness than those in normal tissues, this results in an enhanced uptake of tasquinimod bound to albumin in cancer tissue via a tumor specific process known as the “enhanced permeability and retention” (i.e., EPR) effect. Thus, despite plasma levels of < 1 μM, the EPR effect results in intracellular drug concentrations of 2-3 μM, levels several-fold higher than needed for inhibition of endothelial sprouting (IC_50_ ~ 0.5 μM) or for inhibition of HDAC4 and S100A9 mediated tumor growth.

## INTRODUCTION

**Figure 1:**
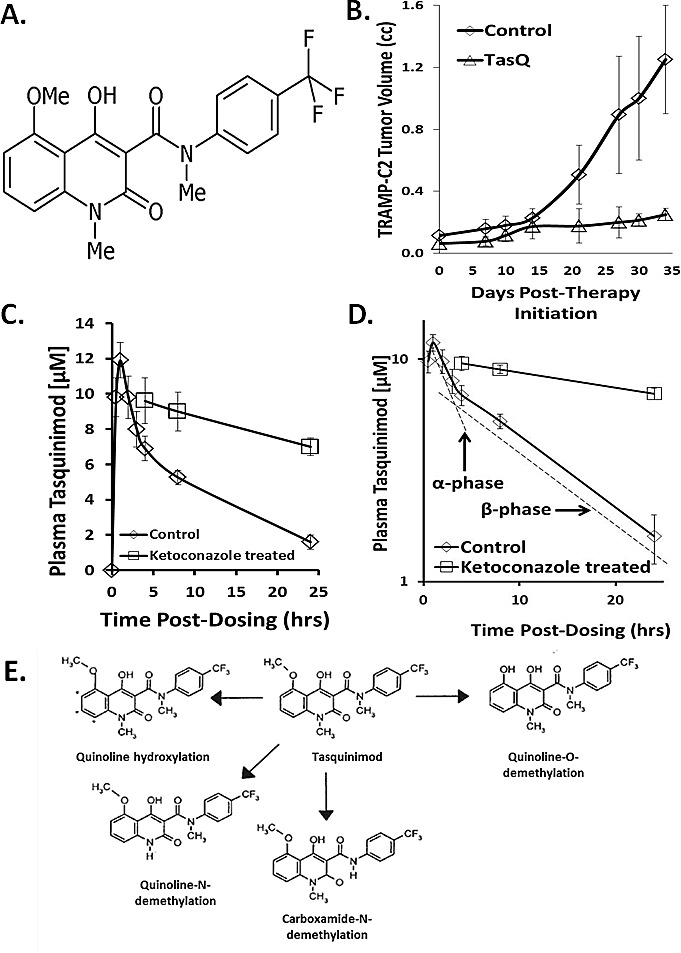
A Chemical structure of tasquinimod B. *In vivo* response of TRAMP-C2 mouse prostate cancers growing in syngeneic C57Bl/6J intact male mice given nothing as controls or given continuous oral dosing with tasquinimod at 5mg/kg/d (i.e., N=10 mice per group). Treatment was initiated when cancers were 100mm^3^. Results presented as mean ± SE. Tasquinimod inhibition is statistically significant (p<0.05) after day 14 of treatment. C. Plasma concentration of tasquinimod after a single oral gavage of 5mg/kg to intact male nude mice given alone or in combination with 25mg/kg/day of ketoconazole (N= 3-5 independent samples). D. Log of plasma concentration of tasquinimod after a single oral gavage of 5mg/kg to intact male nude mice given alone or in combination with 25mg/kg/day of ketoconazole showing the kinetics of the tissue distribution (i.e., α-) phase vs. the elimination (i.e., β-) phase. E. Overview of the metabolism of tasquinimod.

Cancers outgrow their blood supply resulting in the tumor microenvironment becoming acidic, hypoxic, and low in nutrients [1]. To continue growth in such a compromised (i.e., stressful) tumor microenvironment, cancer cells must initiate adaptive survival pathways and activate an angiogenic switch [2]. Activation of this switch recruits infiltrating host cells such as endothelial cells, myeloid- derived suppressor cells (MDSC), macrophages, and bone marrow derived mesenchymal stem cells needed for the chronic stimulation of tumor angiogenesis [2-6]. Tasquinimod (ABR-215050; PubChem CID 546828876; CAS number 254964-60-8), Figure 1A, is an orally active quinoline-3-carboxamide which produces robust and consistent *in vivo* growth inhibition as well as suppression of metastasis in a large series of pre-clinical human xenograft and rodent prostate cancer models [7-12]. Tasquinimod's anti-cancer efficacy involves its inhibition of the reciprocal survival signaling pathways between the cancer cells and tumor infiltrating host cells suppressing the angiogenic switch needed for continued malignant growth in the compromised tumor microenvironment [9- 12]. Tasquinimod is currently in clinical development for the treatment of prostate cancer and other solid malignancies [13, 14]. In a placebo-controlled, phase II randomized trial, tasquinimod doubled median progression-free survival and prolonged survival of patients with metastatic, castrate resistant prostate cancer [15, 16]. A registration phase III clinical trial of tasquinimod as monotherapy in the same patient population is ongoing (NCT01234311).

Mechanistic studies document that tasquinimod's therapeutic response is initiated by high affinity binding to at least two proteins, histone deacetylase 4 (HDAC4) [12] and S100A9 [17, 18]. Binding to HDAC4 is relevant since prostate cancer cells over express this protein and are inhibited in their adaptive survival response to a stressful microenvironment when transcription of this gene is knocked down resulting in their loss of tumorigenic ability [12]. In addition, when HDAC4 is knocked down in endothelial cells, their angiogenic ability is suppressed [12]. Tasquinimod treatment phenocopies HDAC4 knock down with regard to inhibition of prostate cancer cell survival signaling and endothelial angiogenesis under stressful hypoxic conditions [12]. Additional studies document that when tasquinimod binds to the open conformation of HDAC4, it prevents binding of N-CoR/HDAC3 thus preventing client protein deacetylation in both hypoxic cancer cells and their endothelial support cells needed for survival signaling and angiogenesis [12].

In addition to HDAC4, tasquinimod also binds S100A9 [17, 18], a 14 kDa Ca^2+^ and Zn^2+^ binding protein which forms homo- and hetero- (with S100A8) dimers [24]. When secreted, S100A9 undergoes a conformational change becoming an agonist for the pro-inflammatory Toll-like receptor 4 (TLR4) and the receptor for advanced glycation end products (RAGE) inducing proinflammatory danger signals [17]. Tasquinimod binds to S100A9 in a Zn^2+^ dependent manner and thus inhibits TLR4 and RAGE induced signaling [17, 18]. This is significant because S100A9 binding to TLR4 stimulates tumor infiltration of MDSCs [3, 17, 25]. MDSCs in turn express and secrete S100A9 and can, under hypoxia, differentiate via HIF-1α dependent transcription into tumor- associated macrophages which secrete angiogenic factors such as VEGF, FGF, TNF-α, and TGF-β [4, 5]. This is consistent with the observation that exogenous S100A9 can stimulate matrigel plug angiogenesis *in vivo* [26]. Also, an increase in HIF-1α protein induced by hypoxia enhances transcription, expression, and secretion of S100A9 protein in the tumor microenvironment also by prostate cancer cells [27].

Tasquinimod's physiochemical properties and high gastrointestinal permeability contribute to its excellent bioavailability and oral absorption when given at a daily dose either as a liquid or as a gelatin capsule [13]. Once in the blood, quinoline-3-carboxamide compounds like tasquinimod are metabolized primarily by the liver to both more, as well as, less water soluble metabolites [28, 29]. Despite its small size and its metabolism, chronic daily oral dosing with only 1 mg of tasquinimod (i.e., 14.2 μg/kg or 35 nmoles/kg) in clinical trials maintains a blood level of ~ 0.5μM with a plasma half-life of 40 ± 16 hours [13]. The present studies aimed at elucidating the mechanism(s) behind these findings and to explore whether the parent drug or metabolites accounts for the high potency of tasquinimod.

## RESULTS

### Pharmacokinetics (PK) of Tasquinimod

The bioavailability and oral absorption of tasquinimod is excellent when adult male mice (i.e., C57Bl/6J, or athymic nude mice) are given 0.1-30 mg/kg (i.e., 0.2-74 μmoles/kg) via gavage or the drinking water [7]. The potency of tasquinimod expressed as the daily oral dose of tasquinimod which inhibits cancer growth by 50% ranges from 0.1-1.0 mg/kg/d (i.e., 0.24-2.40 μmoles/kg/day) against a series (n>5) of human prostate cancer xenografts in immune-deficient mice [7-12]. Tasquinimod's oral potency is not restricted to human prostate cancer xenografts growing in immune-suppressed mice. For example, tasquinimod at a chronic dose of 5 mg/kg/day via the drinking water produces > 80% inhibition (p<0.05) of TRAMP-C2 mouse prostate cancer growth in immune-competent syngeneic mice, Figure 1B. Associated with this growth inhibition is a 75 ± 9% inhibition (p<0.05) of blood vessel density within the TRAMP-C2 cancers in the tasquinimod treated vs. control animals.

To determine whether this chronic 5 mg/kg/day oral dosing regimen produces similar blood concentrations in mice as obtained in human trials, intact adult male C57Bl/6J mice (n=4) were given ^14^C- radiolabeled tasquinimod via oral gavage and then the plasma concentration of drug at the end of 5 days determined based upon radioactivity levels. These results validated that dosing with 5 mg/kg/day produces a nadir plasma concentration of 1.08 ± 0.21 μM in mice which is the same concentration range as in the human clinical trials. Based upon these results, oral dosing of 5 mg/kg/day was used for all the subsequent PK studies.

To determine the PK of tasquinimod, nude mice with growing (i.e., 200-400 mm^3^) CWR-22Rv_1_ human prostate cancer xenografts were given ^14^C-radiolabeled drug at a dose of 5 mg/kg via oral gavage and the plasma and tissue (i.e., liver, kidney, and tumor) drug concentration determined. Maximum tasquinimod plasma concentration is reached within 1 hour after oral gavage of mice, Figure 1C. After this absorption phase, the plasma concentration declines bi-exponentially as the sum of two first-order processes involving tissue distribution, with the plasma concentration remaining above 1 μM for at least 24 hours, Figure 1D. These PK results support a two-compartment model composed of a central and peripheral compartment. In the absorption phase, the apparent volume of distribution is low (i.e., 0.31 L/kg) but is still more than 3 times the total volume of the blood (i.e., 0.1 L/kg [30]) and is equal to the extracellular body fluid (i.e. 0.2 L/kg [31]) plus the total volume of blood in a mouse (i.e., 0.30 L/kg).

### Metabolism of Tasquinimod

After the rapid α-distribution phase following oral dosing of 5 mg/kg of tasquinimod, there is a much slower β-elimination phase (i.e., half-life > 10 hour), Figures 1C&D. During this β-elimination phase, tasquinimod in the extracellular fluid can enter cells where it can be metabolized. The liver is the major site of metabolism for quinoline-3-carboxamide compounds via Cytochrome P450 3A (CYP-3A) which can be inhibited by ketoconazole [29]. This conclusion is confirmed by the demonstration that co-treatment of mice with ketoconazole, at an oral dose of 25mg/kg/day which inhibits CYP-3A by > 90% [32], decreases tasquinimod's β-elimination phase resulting in an increase in the plasma elimination half-life by more than 8 fold, Figure1D. Metabolically, tasquinimod can be: 1) hydroxylated in the aromatic quinoline scaffold in multiple sites, 2) N-demethylated at the quinoline nitrogen, 3) N-demethylated at the carboxamide nitrogen, or 4) O-demethylated at the quinoline methoxy group, Figure1E.

**Figure 2:**
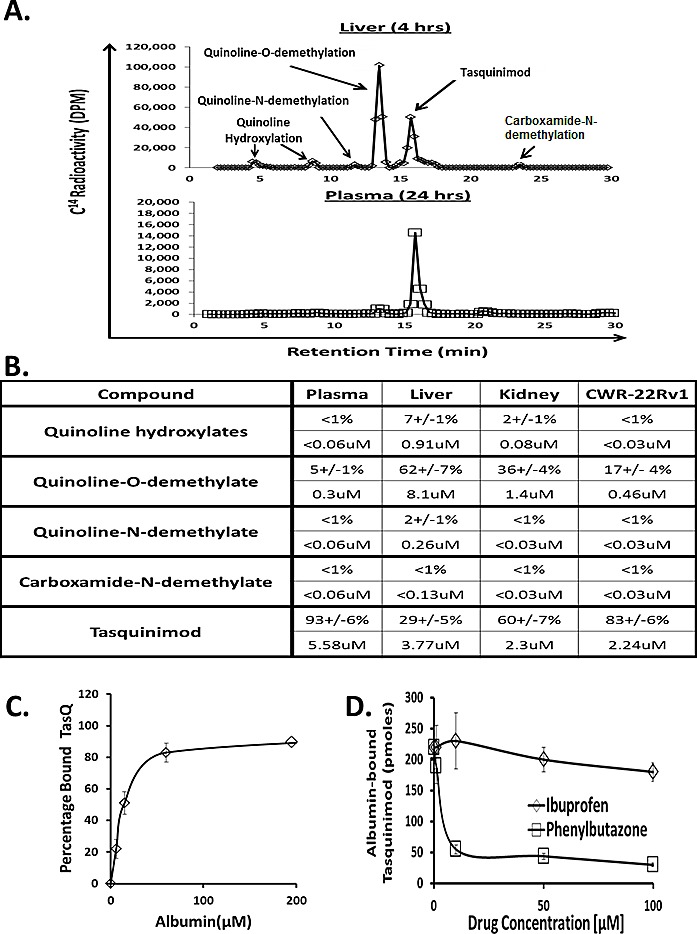
A HPLC separation of the various metabolites of tasquinimod present in liver at 4hours vs. plasma at 24 hours. B. Quantitation of metabolites in plasma, liver, kidney, and CWR-22Rv_1_ cancer 4 hours post oral dosing with 5mg/kg tasquinimod expresses as both percentage of total drug (upper panel) and concentration (lower panel) based upon 1gm of tissue equaling 1ml. (N= 3-5 independent sample per tissue). C. Concentration dependent binding of tasquinimod to human serum albumin. D. Competitive inhibition of tasquinimod binding to human albumin by ibuprofen vs. phenylbutazone. Bicalutamide, like ibuprofen, did not compete with tasquinimod binding (data not shown).

To determine the concentration of metabolites, nude mice bearing CWR-22Rv_1_ prostate cancers were orally administered with 5 mg/kg of ^14^C- radiolabeled tasquinimod and 4 and 24 hours later, blood plasma and liver, kidney, and cancer tissue harvested and analyzed for determination of metabolites. These studies demonstrated that by 4 hours post administration more than 70% of the drug within the liver is present as metabolites, in line with the liver being the major site of metabolism of tasquinimod. The predominant metabolite is the O-demethylated quinoline compound, Figure 2A&B, but the concentration of the hydroxylated aromatic quinoline and the N-demethylated quinoline compounds, along with the O-demethylated quinoline compound are also higher in the liver than the blood at the time point studied, Figure 2B. Also in the kidney 40% of the drug is present as metabolites, with the concentration of the hydroxylated aromatic quinoline and the O-demethylated quinoline compounds being higher than in the blood, Figure 2B. In contrast, within the cancer > 80% of the total concentration of drug is present as parent tasquinimod, Figure 2B. Only the O-demethylated quinoline compound is present at a higher concentration in the cancer than blood, Figure 2B. The plasma concentration of total metabolites is < 10% at 4 hours, Figure 2B, and 24 hours post oral dosing with 5 mg/kg, Figure 2A.

### Plasma Protein Binding of Tasquinimod

Analysis of fractionated whole blood at 4hours post oral dosing indicates that tasquinimod is nearly exclusively (> 98%) located in the plasma and not in blood cells. An explanation for this highly restricted cellular uptake in the blood is provided by the fact that protein binding fraction (PBF) for tasquinimod in nude mouse plasma is 98 ± 1%. This is not a nude mouse specific effect since incubation of 1-50 μM radiolabeled tasquinimod with human plasma or bovine fetal serum (FBS) results in a PBF of ~ 98%.

Using human plasma depleted of albumin identified that the tasquinimod binding protein in the blood is exclusively albumin. Such tasquinimod binding is albumin concentration dependent, Figure 2C. Using equilibrium dialysis, tasquinimod binds to human, mouse, and bovine albumin at pH 7.4 with equilibrium dissociation constant (i.e., K_d_) of 24 ± 4 μM, 16 ± 3 μM and 31 ± 6 μM, respectively. These affinities and the fact that plasma albumin concentration is > 450 μM in all of these species explain why ~ 2% of the total plasma tasquinimod is non-protein bound following oral dosing.

### Albumin Binding Site for Tasquinimod

Albumin has a modular structural organization composed of 3 homologous domains (i.e., domain I, II, and III) each consisting of two separate helical subdomains termed A and B connected by a random coil [33]. Drug binding is via one of three sites with the first located in the IB subdomain, the second the IIA subdomain [i.e., Sudlow's site I] and the third in the IIIA subdomain [i.e., Sudlow's site II] [33]. The antiandrogen, bicalutamide, is a typical IB ligand [34] while phenylbutazone is a typical Sudlow's site I ligand which usually contains bulky heterocyclic molecules with a negative charge located in the middle of the molecule) while ibuprofen is a typical Sudlow's site II ligand which often contains an aromatic carboxylic acid with a negatively charged acidic group at one end of the molecule away from a hydrophobic center [33]. To determine whether tasquinimod binds to any of these sites in albumin, bicalcutamide, phenybutazone, and ibuprofen were tested for their dose-response ability to compete with ^14^C-labeled tasquinimod binding to mouse albumin. Only phenybutazone competed with tasquinimod binding to albumin documenting that tasquinimod binds to the IIA subdomain of albumin (i.e., at Sudlow's site I), Figure 2D.

In additional studies, 200 μg (i.e., 3nmol) of human serum albumin (HSA) was incubated with 400 pmoles of ^14^C-labeled tasquinimod at pH 7.4 so that 50% of the drug is protein bound. This solution was then placed in a dialysis chamber opposite an equal volume of a solution containing 8 mg (i.e., 120 nmoles) of drug free HSA at pH 7.4 separated by a membrane which is permeable to unbound free tasquinimod, but impermeable to albumin. The 8 mg of HSA in the initial drug free side was chosen because this amount of albumin can bind 98% of the drug when exposed to 400 pmoles of tasquinimod. Analysis of the amount of drug in the two compartments after 4 hours of dialysis demonstrated that 65 ± 9 % of the initial protein bound tasquinimod is released demonstrating that tasquinimod binding to albumin is a reversible process.

### Consequence of Tasquinimod Albumin Binding

**Figure 3:**
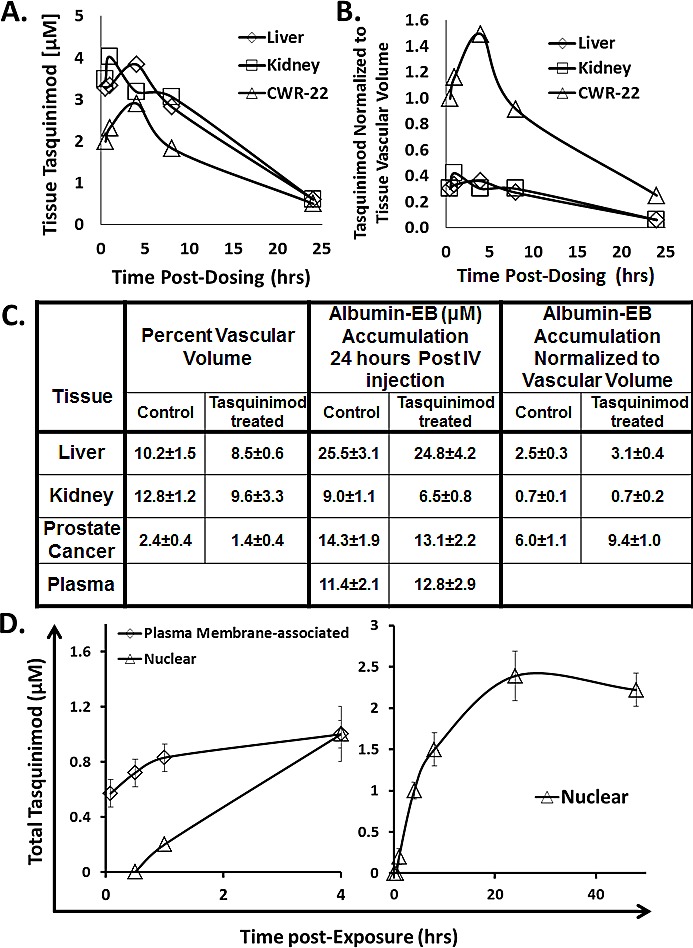
A Concentration of tasquinimod (μM) in indicated tissues at various hours post oral dosing with 5mg/kg of tasquinimod (N= 3-5 independent sample per tissue). B. Tasquinimod tissue concentration normalized per tissue vascular volume at various hours post oral dosing with 5mg/kg of tasquinimod. C. Vascular volume in liver, kidney, and CWR-22Rv_1_ cancer and the accumulation of albumin-Evans Blue 24 hours post IV dye injection expresses as either absolute concentration in μM or normalized to vascular volume. Determinations were performed on 5-7 tumor bearing mice either untreated or given 10mg of tasquinimod/kg/day for 30 days. D. Kinetics of total extracellular plasma membrane-associated concentration vs. the nuclear concentration of tasquinimod by CWR-22Rv_1_ human prostate cancer cells following exposure to 1μM of the drug (N= 3-5 independent sample per tissue).

Following oral dosing with 5 mg/kg, the total plasma C_max_ is ~12 μM, Figure 1C, and thus the C_max_ for protein free-tasquinimod is only ~ 0.24 μM. It is the free tasquinimod which then distributes rapidly into the extracellular fluid of tissues with a half-life for this α-distribution phase of only 3.2 hours, Figure 1D. When CWR-22Rv_1_ tumor bearing mice (n=4) are orally dosed with 5 mg/kg, the total drug concentration in normal tissues (e.g. liver and kidney) and cancer reaches a maximum within the first 5 hours post oral dosing, Figure 3A. When the tissue concentrations are normalized based upon the percent vascular volume in normal (i.e., 10 ± 2% for liver and 13 ± 3% for kidney) vs. cancer tissue (i.e., 2 ± 1% for CWR-22Rv_1_ prostate cancer), prostate cancer tissue has a 3-5 fold higher (p<0.05) concentration of tasquinimod than does either liver or kidney, Figure 3B. These results are consistent with the fact that blood vessels in sites of cancer are known to be much more permeable to albumin than in normal tissues [35, 36]. Such a tumor specific process is known as the enhanced permeability and retention (i.e., EPR) effect [35, 36].

To evaluate the potential importance of an EPR effect for enhancing tumor specific tasquinimod uptake and thus anti-cancer potency, albumin retention in plasma and normal tissue vs. prostate cancer was determined. To do this, nude mice bearing CWR-22Rv_1_ prostate cancers were injected intravenously (IV) with Evans Blue (EB) at a dose (10mg/kg) so that all of the dye is initially bound to albumin within the blood [35]. Twenty-four hours later, the amount of albumin-EB complex retained in plasma vs. accumulated in liver, kidney, or CWR-22Rv_1_ prostate cancers determined. The results are expressed as concentration of extractable albumin-EB in plasma vs. the specified tissue. These studies document that prostate cancers accumulate a concentration of albumin-EB that is equal to that in the plasma by 24 hours, Figure 3C. When normalized to vascular volume, the albumin-EB accumulation by prostate cancers is more than two fold greater (p<0.05) compared to liver and more than 8 fold greater (p<0.05) compared to kidney, Figure 3C. As a follow up to these results, the experiment was repeated using CWR-22Rv_1_ prostate cancer bearing nude mice which were treated with tasquinimod (i.e., 10 mg/kg/ day) for a month. Such continuous treatment with tasquinimod decreased the vascular volume within the cancers by nearly half (p<0.05), Figure 3C. This anti-angiogenic response resulted in a greater than 80% inhibition (p<0.05) in cancer growth. When normalized to this decreased vascular volume, the accumulation of albumin-EB 24 hours post IV injection of the dye in cancers is increased (p<0.05) by 60% compared to non-tasquinimod treated hosts, with no change in normalized accumulation in either the kidney or liver, Figure 3C.

### Tasquinimod Plasma Membrane vs. Nuclear Uptake

Since tasquinimod binds to both S100A9 and HDAC4 proteins with high affinities and since S100A9 has both an extracellular and intracellular location [24] while HDAC4 in prostate cancer cells is in the cell nucleus [12], it is important to evaluate whether tasquinimod is capable of entering cells or whether it only associates with extracellular plasma membrane components. To resolve this question, an *in vitro* approach was taken using three human prostate cancer cells (i.e., CWR-22Rv_1_, LAPC-4, and LNCaP) and human umbilical vein endothelial (HUVEC) cells. When 1 μM tasquinimod is added to 10% fetal bovine serum (FBS) supplemented media, there is sufficient albumin to bind 80% of the drug so that the protein-free concentration of tasquinimod in such media is only 0.2 μM. Preliminary studies documented that even after 48 hours of exposure of HUVEC and each of the 3 prostate cancer cell lines to 1 μM at confluent cell density, there was < 15% metabolism of the parental tasquinimod drug with the major metabolite being the O-demethylated quinoline compound.

Based upon these preliminary validation, cells were incubated in 10% fetal bovine serum (FBS) supplemented media containing 1μM ^14^C-labeled tasquinimod for <10 minutes. When these cells are rapidly (< 20sec) separated from the media by vacuum filtration onto glass microfiber filter and the filters rapidly (< 20sec) washed, radiolabeled drug is retained by the washed cells and this radioactivity can be used to calculate the total cell associated concentration of drug (CA_t_). If instead after the washes, media are added above the filters and allowed to remain for a 15 minutes “wash out” period before being vacuumed off and subsequently rapidly (< 20sec) washed with drug free media, no radioactivity is retained by the washed cells. These results validate that the 15 minute washout period allows elimination (i.e., dissociation) of low affinity drug association with plasma membrane components while retaining the total intracellular concentration of drug (IC_t_). Thus, total plasma membrane associated drug concentration (PM_t_) is determined by subtracting the IC_t_ from the CA_t_ value.

Using this washout protocol, aliquots of CWR-22Rv_1_, LAPC-4, or LNCaP human prostate cancer cells, as well as human umbilical endothelial cells (i.e., HUVECs) were incubated from 5 minutes to 48 hours with 1 μM ^14^C-labeled tasquinimod in 10% FBS media containing 0.2 μM free non-albumin bound drug and the PM_t_ determined. Since essentially identical kinetic results were obtained for all 3 prostate cancer lines as well as for HUVEC cells, only representative results for the CWR-22Rv_1_ cells are presented in Figure 3B, left panel. These results document that the total plasma membrane associated drug concentration (PM_t_) rapidly reaches a steady state value of ~ 1 μM within 5-10 minutes which exceeds that of the protein-free drug concentration (i.e., 0.2 μM) in the media, but is equal to total drug concentration in the media. This ~1 μM PM_t_ is subsequently maintained during the 48 hours observation period.

To determine the total nuclear concentration of drug (N_t_), a modified protocol was needed to allow the rapid lyses of cells and rapid isolation of cell nuclei by differential centrifugation. This could not be accomplished using the usual method of detergent lyses because detergents extract bound drug due to its lipophilicity. Therefore, a detergent free protocol using nitrogen cavitation was developed to rapidly lyse cells and allow the rapid isolation of nuclei via differential centrifugation. Using this cavitation protocol, it was documented that within 1 hour, total nuclear drug concentration (N_t_) equals that of the protein-free drug concentration in the media (i.e., 0.2 μM), and that after 4 hours equals PM_t_ (i.e., see Figure 3B, right panel for representative results for the CWR-22Rv_1_ cells). From 8 hours onward, N_t_ is more than 10 fold higher than the free and 2 fold higher than the total drug concentration in the media, Figure 3B, right panel. For example at 24 hours post exposure to 1 μM tasquinimod in 10% FBS containing media, the N_t_ is 2.4 ± 0.3 μM, Figure 3B, right panel. At this 24 hours period, total radioactive drug in isolated nuclei from all 3 prostate cancer lines, as well as HUVEC cells (i.e., nuclear concentration in these 4 lines ranged from 2.2-3.1 μM) was extracted with acetonitrile and the extracts analyzed by HPLC. These results documented that > 95% (i.e., more than a log higher than any of its metabolites) of the drug present within nuclei from each of these 4 cell lines is non-metabolized tasquinimod. These results raise the issue of whether nuclear uptake of tasquinimod by cancer and endothelial cells requires binding to albumin. To resolve this, nuclear uptake was determined 24 hours post exposure to 1 μM tasquinimod in serum free media. These studies again documented that nuclear tasquinimod concentration in these 4 cell lines ranged from 2.5-3.5 μM documenting that binding to albumin is not required for cell uptake.

### Tasquinimod does not require metabolism for activity

The nuclear uptake results are consistent with tasquinimod being the active drug for both direct therapeutic effects on cancer cells and indirect effects via suppression of the angiogenic response of host endothelial cells. To confirm these conclusions, the concentration of tasquinimod metabolites produced by endothelial cells was compared to the potency of these metabolites for inhibiting angiogenesis. When HUVEC cells are exposed to 1 μM of ^14^C-labeled tasquinimod for 48 hours, < 15% of the drug is metabolized with 10% (i.e., 100 nM) being converted to the quinoline-O-demethylated compound and < 2% (i.e., < 20 nM) being converted to the quinoline-N-demethylated or carboxamide-N-demethylated compounds.

**Figure 4:**
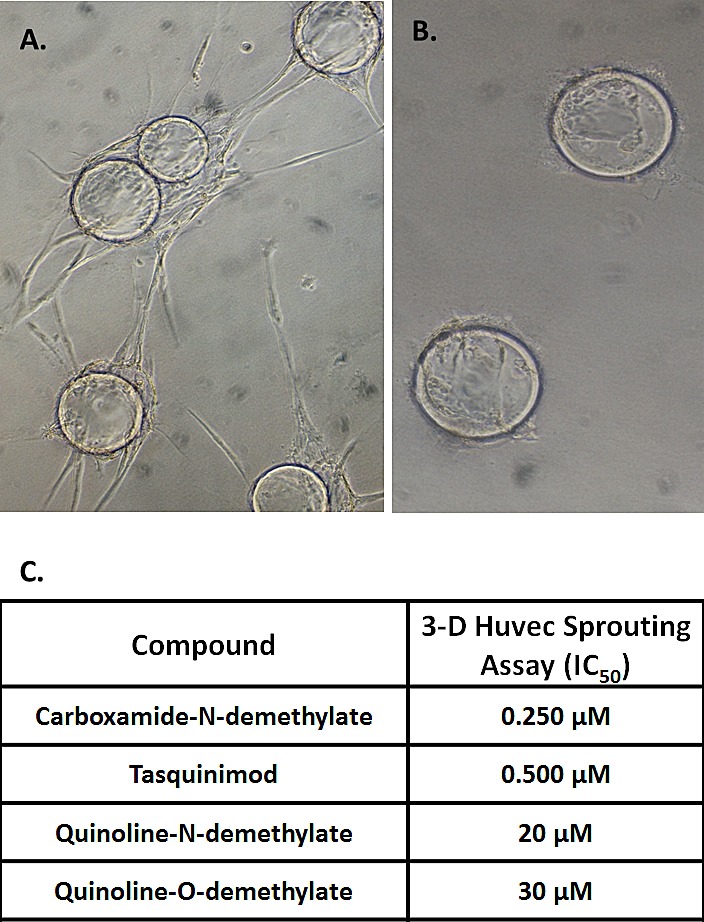
A Sprouting of HUVEC cells in 3-D culture following 5 days of treatment with conditioned media from fibroblasts. B. Lack of sprouting of HUVEC cells in 3-D culture following 5 days of co- treatment with conditioned media from fibroblasts plus 1μM of tasquinimod. C. Potency expressed the μM concentration of the compound needed to inhibit by 50% the sprouting in the 3D-HUVEC assay. (N= 3-6 independent replicate per assay with 3-5 independent assays per compound).

A three-dimensional (3D) *in vitro* endothelial sprouting and tube formation assay was utilized to define the anti-angiogenic potency of tasquinimod vs. these metabolites. In this 3D assay, human umbilical endothelial cells (i.e., HUVECs) are initially grown on microcarrier beads and the cell coated beads embedded in fibrin gels with media conditioned by normal human lung fibroblasts [12]. Under these 3D-conditions, endothelial cells “sprout” producing 3D neovessel tubes with patent lumen within one week, Figure 4A - left panel. When tasquinimod is added at the initiation to these 3D-cultures, sprouting of the endothelial cells is inhibited, Figure 4B-right panel, with the potency for such inhibition, expressed as an IC_50_ value (i.e., the concentration of drug which inhibits by 50% the endothelial sprouting) of 0.5 μM, Figure 4C. These results are significant since in clinical trials, tasquinimod's systemic anti-cancer potency coincidently occurs at plasma levels of 0.5-1 μM [13, 15,]. Using this 3D-HUVEC assay, the potency (i.e., expressed as IC_50_) of tasquinimod metabolites to inhibit endothelial sprouting was compared to tasquinimod. These results document that only the carboxamide-N-demethylated metabolite is more potent (i.e., 2 fold lower IC_50_), with the other metabolites being 40-60 fold less potent (i.e., higher IC_50_) than tasquinimod, Figure 4C. Combining these sprouting results with the HUVEC tasquinimod metabolic studies documents that the concentration of carboxamide-N-demethylated metabolite following exposure to 1 μM tasquinimod (i.e., < 20 nM) is more than a log below its IC_50_ and thus despite being 2-fold more potent than tasquinimod, it is not the major active moiety. To further confirm that tasquinimod itself and not the carboxamide-N-demethylated metabolite is the active drug, the response to ketoconazole alone and in combination with tasquinimod was tested in the 3D-endothelial sprouting assay. These studies demonstrate that ketoconazole alone did not inhibit endothelial sprouting even at a concentration of 10 μM which is > 50 times the IC_50_ of 0.2 μM for ketoconazole inhibition of CYP-3A [29]. In addition when 10 μM ketoconazole is simultaneously present, it does not prevent inhibition of 3D HUVEC sprouting produced by 1 μM tasquinimod.

**Figure 5:**
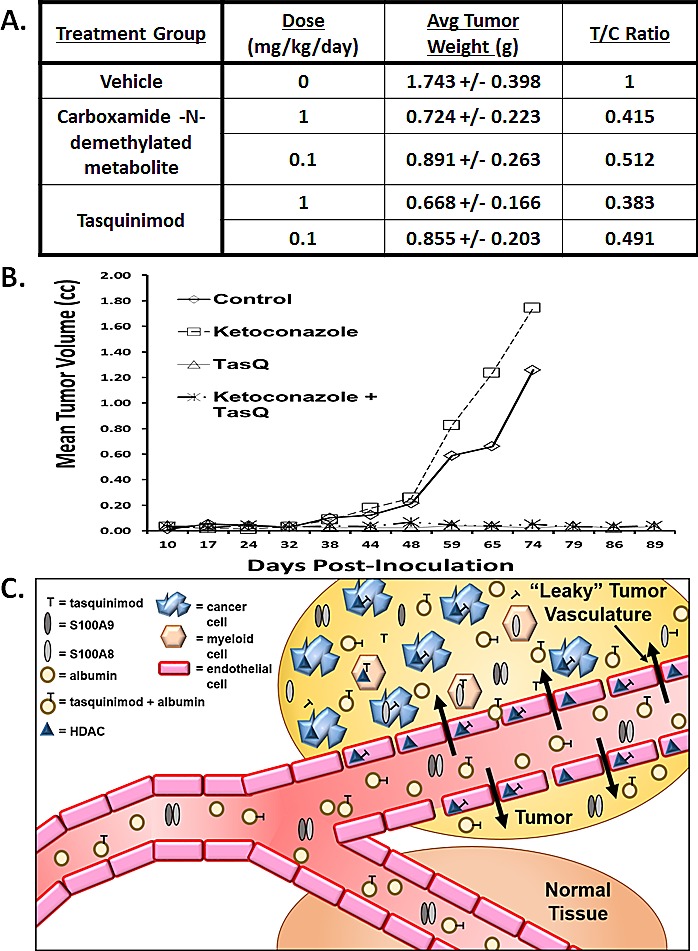
A Dose-response growth inhibition induced by tasquinimod vs. its carboxamide-N- demethylated metabolite when given for 20 days via subcutaneous daily injection to intact adult male nude mice bearing established CWR-22Rv_1_ human prostate cancer xenografts. Treatment was started when the cancer were ~200mm^3^ (N=8-10 animals per group). Both compounds at both doses produced significant (p<0.5) tumor growth inhibition. B. Inhibition of tumor take of CWR-22RH xenografts in castrated adult male nude mice induced by chronic oral treatment with either nothing (i.e., control), tasquinimod alone (i.e., 10mg/kg/d via drinking water), ketoconazole alone (i.e., 25mgs/kg/d via oral gavage) or tasquinimod plus ketoconazole. Treatment was started at the day of tumor inoculation (N=8-10 animals per group). C. Proposed mechanism for the delivery of albumin bound tasquinimod which accumulates due to the coupling of leaky blood vessels and a lack of lymphatic drainage characteristic within the tumor microenvironment (i.e., EPR effect) and its uptake of free drug into myeloid-, endothelial- and tumor cells.

To confirm these *in vitro* results, mice were inoculated subcutaneously (SQ) with CWR-22Rv1 cells and remained untreated until the tumors grew to ~ 200 mm3 (i.e., ~ 200 mgs) and then they were randomly assigned to groups (n=10) which were injected SQ daily with 100 μl of propylene glycol vehicle only vs. vehicle delivering 0.1 or 1 mg/kg/day of either tasquinimod or its carboxamide-N-demethylated metabolite. Propylene glycol was used as vehicle due to the low water solubility of the carboxamide-N-demethylated metabolite. After 20 days of continuous treatment, the experiment was terminated and the total weight of each cancer determined and the results expressed both as average tumor weight in grams and as the ratio of the weight of the cancers in the treated vs. vehicle control animals (i.e., T/C ratio), Figure 5A. These results demonstrated that on an equal dose basis, tasquinimod is equipotent to its carboxamide-N-demethylated metabolite which speaks against metabolism being required for antitumor activity *in vivo*.

To confirm this conclusion, castrated male nude mice were xenografted with the castrate resistant CWR-22RH human prostate cancer tissue and the animals immediately randomized into groups (n=10) which were: 1) left untreated as controls; 2) given daily oral treatment with tasquinimod alone via the drinking water (10mg/kg/day); 3) given oral ketoconazole alone via oral gavage at a daily dose which inhibits cytochrome P450 3A4 (i.e., 25 mg/kg/day) [32]; or 4) given a combination of the two drugs. The ability of this ketoconazole dosing regimen to inhibit tasquinimod metabolism was validated by the demonstration that it prolong (p<0.05) the plasma clearance of tasquinimod, Figures 1B&C. These results document that if chronic tasquinimod treatment was initiated simultaneously with inoculation, instead of initiating treatment when the cancers were already established and growing, tasquinimod prevents tumor take and subsequent malignant growth, Figure 5B. In contrast, ketoconazole alone at a dose which inhibits tasquinimod metabolism when given by itself does not inhibit either the take or growth of the CWR-22RH human prostate cancer, and when simultaneously combined with tasquinimod, it does not inhibit the therapeutic efficacy of tasquinimod to prevent initial tumor take, Figure 5B. These combined *in vitro*/*in vivo* results document tasquinimod, not one of its metabolites, is the active drug.

## DISCUSSION

Chronic once daily oral dosing with tasquinimod doubles the median progression-free survival and prolongs survival of patients with metastatic, castrate resistant prostate cancer in randomized prospective clinical trials [15, 16]. These positive clinical results are produced using an oral dose of tasquinimod of only 0.5-1 mg/day. On a body weight basis, this translates into a daily dose of only 7-14 μg/kg/day (i.e., 17-34 nmol/kg/day) which maintains a plasma level of tasquinimod of ~ 0.5 μM with a plasma half-life of 40 hours [13]. Tasquinimod can be metabolized via CYP-3A4, Figure 1 C&D, to at least one other compound (i.e., the carboxamide-N-demethylated metabolite) which is 2-fold more potent in inhibiting endothelial cells sprouting *in vitro*, Figure 4C. However, the extent of production of this metabolite is too minor for it to be the active drug, Figure 2B, since detectable levels of this metabolite could not be found in the endothelial and cancer cells responding to tasquinimod treatment either *in vitro* or *in vivo*. This conclusion is further supported by the observation that simultaneous combination treatment with a dose of ketoconazole which completely inhibits CYP-3A4 induced tasquinimod metabolism does not suppress tasquinimod's ability to inhibit endothelial “sprouting” *in vitro* or anti-cancer efficacy against human prostate cancer xenografts *in vivo*, Figure 5B. These combined *in vitro/in vivo* results document that tasquinimod, not one of its metabolites, is the active drug.

The present studies also identified that tasquinimod reversibly binds to the IIA subdomain of albumin (i.e., Sudlow's site I) with an equilibrium dissociation constant of less than 30 μM, Figure 3C&D. This affinity together with a plasma albumin concentration of > 450 μM [37], explains why only ~ 2% of the total plasma tasquinimod is non-protein bound following oral dosing. The fact that 98% of the plasma tasquinimod is albumin bound has mechanistic consequences for its high potency as an anti-cancer drug. While the half-life of albumin is ~ 19 days in humans, its plasma half-life is only about 1 day [37]. This is because despite tight endothelial barrier function, albumin constantly leaves the plasma via endothelial transcytosis and enters the extracellular fluid throughout the body [38]. Due to this transcytosis, albumin makes ~ 15 around trips during its life-span to the extracellular fluid returning via the lymphatic thoracic duct back to the blood [37]. A distinct characteristic of cancer tissue is a decrease in tumor endothelial barrier function which enhances the leakage of plasma macromolecules like albumin into the extracellular fluid which is coupled with a lack of a functional lymphatic drainage [39]. Thus, unlike the situation in normal tissues where albumin returns from the extracellular fluid to the plasma via lymphatic drainage, once in cancer extracellular fluid, albumin is trapped and does not re-enter circulation [38].

A drug that interacts sufficiently strong with albumin may be concentrated within the cancer tissue through the tumor specific process known as the enhanced permeability and retention (i.e., EPR) effect [35, 36]. As demonstrated by the present studies, tasquinimod binds to albumin with an affinity sufficient to result in such an EPR effect. Thus despite its metabolism and limited tumor vascular volume, an oral dose of tasquinimod producing a nadir plasma concentration of only 0.5-1 μM albumin-bound tasquinimod will result in cancer tissue levels in the same range due to the EPR effect, Figure 3A&B. Achieving these levels of the drug is critical because the concentration necessary for inhibiting endothelial sprouting was found to be in this range (IC_50_ ~ 0.5 μM), Figure 4C.

Besides tumor endothelium becoming leaky, as cancers outgrow their blood supply, the tumor microenvironment becomes acidic, hypoxic, and low in nutrients [1]. In this compromised (i.e., stressful) tumor microenvironment, cancer cells initiate adaptive survival pathways and activate an angiogenic switch [2]. Activation of this switch by the cancer cells recruits infiltrating host cells such as endothelial cells, myeloid- derived suppressor cells (MDSCs), macrophages, and bone marrow derived mesenchymal stem cells needed for the chronic stimulation of tumor angiogenesis and cancer cell survival signaling in such a stressful tumor microenvironment [2-6]. Such tumor angiogenesis requires HDAC4 and S100A9 dependent signaling in the cancer cells themselves as well as in the infiltrating host cells [12, 18, 26]. Hence, exposure of cancer and endothelial cells to 0.5-1μM concentrations of tasquinimod will produce intracellular levels of 2 to 3 μM or more than twenty-fold higher than needed to bind and thus also inhibit HDAC4 and S100A9 [12, 18]. In summary, as schematically illustrated in Figure 5C, the proposed mechanism for tasquinimod's potency involves EPR facilitated delivery of albumin bound drug via leaky vessels into the tumor microenvironment lacking lymphatic drainage and the resulting uptake of free compound into myeloid, endothelial and tumor cells inhibiting HDAC4/S100A9 signaling uniquely activated and required for survival and growth within the cancer, but not in normal, tissue microenvironment.

## MATERIAL AND METHODS

### Reagents

Ketoconazole and bicalutamide (i.e., casodex) were obtained from LKT Laboratories Inc. (St Paul, MN). Unlabeled tasquinimod and ^14^C-tasquinimod labeled in the 3 position of the quinoline nucleus at a specific activity of 33.9 mCi/mmol (> 90% purity) were provided by Active Biotech Inc. AB. Tasquinimod metabolites were prepared at Active Biotech AB (Lund, Sweden) as previously described [7, 12].

### *In Vivo* Cancer Growth Inhibition and Anti-Angiogenic Assays

The source, history, and characteristics of the human prostate cancer cell lines (i.e., LNCaP, LAPC4, and CWR-22Rv_1_ and the human umbilical vascular endothelial cells (HUVEC) used, as well as cell culture conditions for their *in vitro* maintenance and the *in vivo* protocol for their xenografting into immune-deficient nude male mice are as described previously [7, 12]. The source, history, and characteristics of the TRAMP-C2 mouse prostate cancer line, as well as cell culture conditions for their *in vitro* maintenance and the *in vivo* protocol for their growth in intact adult male syngeneic C57Bl/6J are as described previously [7]. All lines were mycoplasma negative using the MycoSensor PCR Assay kit (Agilent Technologies) and genetically authenticated within the last 6 months using short tandem repeat profiling conducted by the Johns Hopkins Genetic Resource Core Facility. The source, history, and characteristics of the CWR-RH human prostate cancer xenograft are as described previously [8]. *In vitro* growth curves were determined as described [7, 12]. Animal studies were conducted according to animal protocol MO09M434 approved by Johns Hopkins Animal Care and Use Committee. Daily drug dosing via oral gavage, the drinking water, or subcutaneous injection, as well as tumor volume measurements and determination of the blood vessel density within cancers, were as described previously [7]. These experiments were repeated at 3 independent times for each xenograft.

### Measurement of Vascular Volume

Tissue vascular volumes for liver, kidney, and CWR-22Rv_1_ cancers were determined previously via intravenous infusion of^51^ Chromium (Perkin Elmer, Shelton, CT) labeled red blood cells and the result expressed as percent vascular volume as described previously [10].

### Tasquinimod Plasma Pharmacokinetic and *in vivo* Tissue and *in vitro* Cell Metabolism Assays

Male nude mice bearing 200-300mm^3^ CWR-22Rv_1_ cancers were given ^14^C-labeled tasquinimod at a dose of 5 mg/kg via oral gavage. At times ranging from 30 min to 24 hour, groups of 4-6 mice were anaesthetized with ketamine and 1 ml of heparinized blood collected by cardiac puncture, followed by subsequent cardiac perfusion of saline to remove blood associated drug. An aliquot of liver, kidney, and cancer was removed, weighed and then homogenized. The radioactivity in 10 μL aliquots of tissue homogenates and blood plasma was determined using a liquid scintillation counter and used to calculate the total amount of tasquinimod plus its metabolites per gram of tissue or per ml of blood. The remaining sample volume was deproteinated with acetonitrile and the radioactivity of an aliquot of the supernatant determined and the remaining supernatant lyophilized, resuspended initially with 0.1mL dimethyl sulfoxide and diluted by the addition of 0.9mL deionized water prior to injection into the HPLC. The HPLC system consisted of Waters pumps using a Waters gradient controller to deliver mobile phase at a flow rate of 1mL per minute using a Supelcosil ABZ+ C_18_ column that is 150mm long by 4.6mm diameter and has 5μm particle size. The metabolites were separated using a linear gradient in which the starting mobile phase was 35% acetonitrile: 65% water: 0.1% Trifluoroacetic acid (TFA) increasing to 50% acetonitrile: 50% water: 0.1%TFA over 25 minutes, and then increased rapidly to 98% acetonitrile: 2% water: 0.1%TFA and held until 33 minutes before it is returned to the initial conditions for the next run. Fractions were collected and the radioactivity determined by scintillation counting. As controls, the individual elution time of non-radiolabeled standard solutions of the various tasquinimod metabolites was determined using UV absorption at 215nm. The results were expressed as concentration based upon tissue volume, assuming 1gram wet weight equals 1ml, or normalized to tissue vascular volume.

For *in vitro* studies, cells were incubated with 1μM ^14^C-labeled tasquinimod for indicated time period and then the media and cells or cell nuclei separately harvested, deproteinated with acetonitrile, and metabolites separated by HPLC and quantitated as described above.

### Determination of Protein Binding Fraction (PBF)

Mice were orally gavaged with ^14^C-labeled tasquinimod at a dose of 5mg/kg and 1 hour later blood collected and plasma isolated and the PBF determined as described previously [40] using Centrifree® YM-30 centrifuge filter unit with a molecular weight cutoff of 30K (i.e., Millipore, Billerica,MO).

### Determination of Dissociation Constant for Binding to Albumin

To determine whether albumin is the tasquinimod binding protein in plasma, albumin was removed from human plasma using an albumin depletion kit (Thermo Scientific cat # 85160). The albumin depleted plasma was then tested for its protein binding as described above. The dissociation constant (k_d_) for tasquinimod binding was determined based upon equilibrium dialysis of a mixture of 4mg/ml of the species (i.e., human, bovine, and mouse) specific albumin obtained from Sigma with 2μM of ^14^C-labeled tasquinimod in PBS pH 7.4 buffer using a 96-well equilibrium dialyzer™ (Harvard Apparatus, Holliston,MA).

To identify the binding site of tasquinimod to albumin, the dose-response ability of a typical albumin domain IB ligand (i.e., bicalutamide [33]) vs. a typical domain IIB ligand (i.e., phenylbutazone [32]) vs. a typical site II ligands (i.e., ibuprofen [32]) to compete with ^14^C -labeled tasquinimod binding to mouse albumin was determined using equilibrium dialysis as described above.

### Determination of the Enhanced Permeability and Retention (EPR) Effect

EPR effect was determined using Evans Blue as described previously [34] with the modification that 24 hours after the albumin in the blood of tumor bearing nude mice was labeled via IV injection of Evans Blue (EB) at a dose of 10mg/kg, the animals were anesthetized, blood collected, and then animals were perfused with saline to remove blood from the host tissues before harvesting liver, kidney, and prostate cancer xenograft tissue. The results are expressed as the concentration of EB-labeled albumin retained in the blood or specified tissue.

### Cellular Tasquinimod Uptake

A rapid filtration method was developed and validated to measures the kinetics of tasquinimod cell uptake on a series of human cell lines. To do this, exponentially growing cells in a tissue culture flask are trypsinized, and the number of single cells counted and their average diameter determined electronically using Cellometer AutoT4 (Nexcelom, Lawrence, MA). Then multiple replicates (N=3-6) of 10^7^ cells per time point were incubated from 5 minutes to 48 hours. at 37°C in 1ml of tissue culture media containing 10% fetal bovine serum (FBS) plus 1μM ^14^C-labeled tasquinimod. The cells in each replicate incubation were then rapidly separated from the tasquinimod containing media by vacuum filtration onto a 24mm Whatman GF/C glass microfiber filter (GE Healthcare Sciences, Piscataway, NJ) using a Millipore 1225 sampling manifold (Millipore, Billerica MA). The filters are then rapidly washed and filtered (<20 seconds per wash) 3 times with 2mls of drug free FBS containing tissue culture media. Half of the replicate filters are then dried overnight and counted for determination of the total cell associated amount of drug (i.e., plasma membrane associated plus intracellular uptake). To the remaining half of the replicate filters, 2mls of drug free FBS containing tissue culture media is added above the filters and allowed to remain there for 15 minutes before being filtered off. This “wash off” treatment to remove cell surface associated while retaining intracellular drug is followed by three additional 2ml washes before the filters are dried overnight and counted for the determination of the total intracellular amount of drug. As controls, sham filters without the presence of cells are treated and processed similarly through the multiple washes and then dried and counted. The appropriate control counts are subtracted from both the whole cell and intracellular counts. These corrected radioactivity values are then converted into tasquinimod amount which are then divided by the total volume of 10^7^ cells (i.e., determined using the average cell diameter for each cell line) to determine the total cell associated concentration of drug [CA_t_] vs. total intracellular amount of drug (IC_t_) at the indicated time point. The total plasma membrane associated drug concentration (PM_t_) is determined by subtracting the IC_t_ from the CA_t_ value.

To specifically determine the concentration of tasquinimod within the cell nucleus, 10^7^ cells were incubated in 1ml of tissue culture media containing 10% fetal bovine serum (FBS) plus 1μM ^14^C-labeled tasquinimod for varying times before the cells were centrifuged rapidly (i.e., 1000xG for 2 min) to remove the drug containing media. The cells were rapidly resuspended in phosphate buffered saline and exposed to pressurized (i.e., 500lbs/sq in) N_2_ in a nitrogen cavitation devise (i.e., Parr bomb, Parr Instrument Company, Moline, Il) for 5 min before the plasma membranes were lysed by the rapid release of the pressure. This detergent free procedure lyses >98% of the cells without disrupting either mitochondria or lysosomes. The lysate was then centrifuged at 800Gx 2 minutes to restrictively pellet only cell nuclei. The radioactivity of this pellet was counted and used to calculate the total amount of tasquinimod associated with 10^7^ cell nuclei. To determine the nuclear drug concentration, this total nuclear drug amount was driving by the total volume of 10^7^ cell nuclei determined from the average diameter of the nuclei determined electronically using Cellometer AutoT4.

### *In Vitro* Anti-angiogenesis Assay

The 3-dimensional (3D) *in vitro* angiogenic sprouting and tube formation assay was used as described previously [12]. This 3D-endothelial sprouting assay was conducted using a minimum of 5 replicate wells per drug dose per experiment, repeated 3 independent times using human umbilical vein endothelial cells (HUVECs; Lonza Walkersville, Inc.) with modification that 20 μL of 10X concentrated Dulbecco's Modified Eagle's Media/10%FCS media conditioned by confluent human primary lung fibroblasts (Lonza) diluted to 200 μL of growth factor supplemented EGM-2 media (Lonza) was used instead of fibroblast co-culture.

### Statistics

All of the values reported are presented as means ± SE of representative data generated from 1 of a minimum of 3 independent experiments in which there were a minimum of 5 replicates per data point. Statistical analysis was conducted by a 1-way ANOVA with the Newman- Keuls test for multiple comparisons with significance being p<0.05.
